# Noninvasive Diagnostic Method to Objectively Measure Olfaction and Diagnose Smell Disorders by a Molecularly Targeted Fluorescence Imaging Agent

**DOI:** 10.2967/jnumed.123.266123

**Published:** 2024-08

**Authors:** Dauren Adilbay, Junior Gonzales, Marianna Zazhytska, Paula Demetrio de Souza Franca, Sheryl Roberts, Tara D. Viray, Raik Artschwager, Snehal Patel, Albana Kodra, Jonathan B. Overdevest, Chun Yuen Chow, Glenn F. King, Sanjay K. Jain, Alvaro A. Ordonez, Laurence S. Carroll, Stavros Lomvardas, Thomas Reiner, Nagavarakishore Pillarsetty

**Affiliations:** 1 Department of Radiology, Memorial Sloan Kettering Cancer Center, New York, New York;; 2 Department of Surgery, Memorial Sloan Kettering Cancer Center, New York, New York;; 3 Mortimer B. Zuckerman Mind, Brain and Behavior Institute, Columbia University, New York, New York;; 4 Department of Otorhinolaryngology and Head and Neck Surgery, Federal University of São Paulo, São Paulo, Brazil;; 5 Department of Genetics and Development, Columbia University Irving Medical Center, Vagelos College of Physicians and Surgeons, Columbia University, New York, New York;; 6 Department of Otolaryngology—Head and Neck Surgery, Columbia University Irving Medical Center, Vagelos College of Physicians and Surgeons, Columbia University, New York, New York;; 7 Institute for Molecular Bioscience, University of Queensland, St. Lucia, Queensland, Australia;; 8 Australian Research Council Centre of Excellence for Innovations in Peptide and Protein Science, University of Queensland, St. Lucia, Queensland, Australia;; 9 Center for Infection and Inflammation Imaging Research, Johns Hopkins University School of Medicine, Baltimore, Maryland;; 10 Department of Pediatrics, Johns Hopkins University School of Medicine, Baltimore, Maryland;; 11 Russell H. Morgan Department of Radiology and Radiological Sciences, Johns Hopkins University School of Medicine, Baltimore, Maryland; and; 12 Department of Radiology, Weill Cornell Medical College, New York, New York

**Keywords:** optical, anosmia, COVID-19, fluorescence imaging, olfaction, smell

## Abstract

Despite the recent advances in understanding the mechanisms of olfaction, no tools are currently available to noninvasively identify loss of smell. Because of the substantial increase in patients presenting with coronavirus disease 2019–related loss of smell, the pandemic has highlighted the urgent need to develop quantitative methods. **Methods:** Our group investigated the use of a novel fluorescent probe named Tsp1a-IR800_P_ as a tool to diagnose loss of smell. Tsp1a-IR800_P_ targets sodium channel 1.7, which plays a critical role in olfaction by aiding the signal propagation to the olfactory bulb. **Results:** Intuitively, we have identified that conditions leading to loss of smell, including chronic inflammation and coronavirus disease 2019, correlate with the downregulation of sodium channel 1.7 expression in the olfactory epithelium, both at the transcript and at the protein levels. We demonstrated that lower Tsp1a-IR800_P_ fluorescence emissions significantly correlate with loss of smell in live animals—thus representing a potential tool for its semiquantitative assessment. Currently available methods rely on delayed subjective behavioral studies. **Conclusion:** This method could aid in significantly improving preclinical and clinical studies by providing a way to objectively diagnose loss of smell and therefore aid the development of therapeutic interventions.

## Visual Abstract

**Figure:**
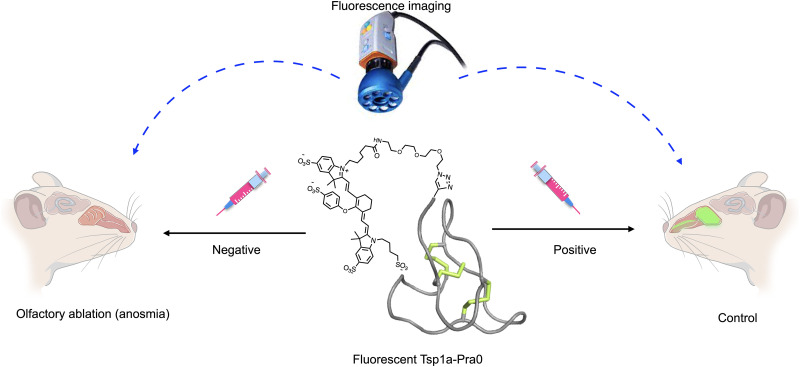

Olfaction (sense of smell) is crucial for the survival of most animals, including humans, attributed to its vital transfer of information about the food and environment, which instinctively serves as a tool for inter- and intraspecies communication (1). Over the past 3 y, mostly as a result of the coronavirus disease 2019 (COVID-19) pandemic, loss of smell (anosmia) has captured the attention not only of the scientific community but also of the general public and has highlighted the need for a deeper mechanistic understanding of this sense. Most importantly, the lack of rapid assessments has exposed the need for objective tools to assess anosmia (2*,*^3^). It is estimated that about 13.3 million adults in the United States have a vast range of smell disorders and that 3.4 million endure severe hyposmia or complete anosmia (4). These studies were performed before the COVID-19 virus pandemic and therefore severely underestimate people currently with smell disorders (5–7).

Despite the fundamental importance of the sense of smell in the quality of life and the high prevalence of anosmia, no quantitative method to assess the perception of smell is currently available either clinically or for use in human or research animal settings. In humans, the perception of smell is subjectively reported and registered as a nonindependent measure. In animals, we rely on behavioral studies, such as the buried-food test, which measures time to find food as negatively correlated with the sense of smell (8). These tests not only are challenging to perform but also are indirect and highly subjective. Developing new diagnostic methods is, therefore, a clinical and scientific need.

Voltage-gated sodium channels (ranging from Na_V_1.1 to Na_V_1.9) are crucial in neurotransmission and aid in the propagation of an action potential, and therefore neurologic impulses, along the nerve bundles. Na_V_1.7 is encoded by the SCN9A gene and is expressed predominantly in the peripheral sensory neurons. Its dysfunction has been correlated with impaired olfaction (9*,*^10^). It is highly expressed in the axons of human olfactory sensory neurons (OSNs), where it plays a critical role in transmitting olfactory cues provided by olfactory receptor activation to higher-order neurons in the brain (10). Dysfunction of Na_V_1.7, originating from mutations in the SCN9A gene or loss of Na_V_1.7 channels at the level of the olfactory bulb and olfactory epithelium, results in smell disorders (10). The presence of Na_V_1.7 in the superficial layer of the olfactory epithelium presents an unprecedented opportunity to develop optic probes that can noninvasively image the expression of Na_V_1.7. By objectively measuring changes in Na_V_1.7 expression in the olfactory epithelium, we can potentially identify smell disorders immediately.

Taking advantage of a potent and exquisite Hsp1a peptide (Supplemental Fig. 1; supplemental materials are available at http://jnm.snmjournals.org), which was isolated from a Peruvian tarantula and targets solely Na_V_1.7 (11–13), our group has previously developed fluorescent and PET imaging agents that can aid visualization of peripheral nerves in vivo without any side effects (12–15). For this work, amenable Tsp1a-Pra0 was derived from Hsp1a and was the starting compound for Tsp1a-IR800_P_ (Supplemental Fig. 1). A fluorescent peptide is used to demarcate olfactory epithelium and Na_V_1.7 changes triggered by inflammation. As a model for smell disorders (allergy, viral infection, etc.), we have used methimazole injections to induce inflammation in the nasal cavity (16). After intraperitoneal methimazole administration, various cell types in the olfactory epithelium, including OSNs and sustentacular cells, exhibit swollen organelles within 4 h, which is subsequently followed by detachment of cells from the basal lamina. In the current article, we establish a robust correlation between anosmia resulting from methimazole treatment or COVID-19 and a decrease in Na_V_1.7 expression in the superficial layer of the olfactory epithelium of mice, hamsters, primates, and humans, and we demonstrate the utility of Na_V_1.7-targeted fluorescent Tsp1a peptides in detecting the changes in Na_V_1.7 expression in OSNs. Our findings suggest that our fluorescent Tsp1a peptide has the potential to serve as a noninvasive imaging tool for detecting loss of smell.

## MATERIALS AND METHODS

### General

Acetonitrile of high-performance liquid chromatography (HPLC) grade and liquid chromatography–mass spectrometry grade was purchased from Fisher Scientific. Water (>18.2 MΩcm^−1^) was obtained from an AlphaQ Ultrapure water system. Reverse-phase HPLC purifications were performed on a Shimadzu HPLC system equipped with a DGU-20A degasser, an SPD-M20A ultraviolet detector, an LC-20AB pump system, and a CBM-20A communication bus module using a reverse-phase HPLC column (Atlantis T3 C18, 5 μm, 4.6 × 250 mm, Waters product number 186003748). Regarding the HPLC solvents, buffer A was H_2_O plus 0.1% trifluoroacetic acid, and buffer B was acetonitrile plus 0.1% trifluoroacetic acid. HPLC purification and analysis were performed at a flow rate of 1 mL/min with a gradient of 5%–95% B for 60 min. Electrospray ionization mass spectroscopy spectra were recorded on a Waters Aquity ultra-performance liquid chromatograph with an electrospray ionization single-quadrupole detector. Compounds were lyophilized on a Labconco FreeZone 2.5 plus. The concentration of Tsp1a-IR800_P_ was determined by measuring the absorbance. Details are provided in Supplemental Figure 1. Regarding the solvents for the mobile phase for the liquid chromatography–mass spectrometry, buffer A was H_2_O plus 0.05% formic acid, and buffer B was acetonitrile plus 0.05% formic acid. The liquid chromatography–mass spectrometry analysis was performed at a flow rate of 1 mL/min with a gradient of 5%–95% B for 15 min.

### Synthesis of Tsp1a-Pra0 and Fluorescent Tsp1a-IR800_P_

On the basis of our previous success with the Hsp1a peptide, we prepared a synthetic version named Tsp1a that incorporates a propargylglycine at the N terminus (Tsp1a-Pra0) to facilitate the click chemistry, following protocols similar to what has been reported before (12–14). Our Tsp1a—which features a propargylglycine—was used to conjugate the peptide to IR800 azide via copper-catalyzed click chemistry to afford Tsp1a-IR800_P_. IR800cw azide dye (50 μg, 44 nmol, LI-COR BioSciences product number 929-60000, in 50 μL of acetonitrile) was conjugated to Tsp1a-Pra0 (propargylglycine modified at the N terminus) peptide (0.72 mM, 250 μg in 100 μL of H_2_O) in a buffer. The Tsp1a-Pra0 peptide was diluted with an aqueous Tris-buffered solution (25 mM, pH 7.4); further, L-ascorbic acid in H_2_O (50 mM), CuSO_4_ solution in H_2_O (50 mM), and IR800cw azide (50 μg, 44 nmol) were added to the reaction mixture. The reaction mixture was stirred at room temperature in the dark for 4 h. The crude mixture was purified via reverse-phase HPLC, fractions containing the product were pooled and lyophilized, and pure Tsp1a-IR800_P_ was obtained as a blue solid (44 μg, 10 nmol; 26% yield). The final HPLC analysis for the Tsp1a-IR800_P_ showed 96% purity after the isolation. On liquid chromatography–mass spectrometry (electrospray ionization–positive), the mass-to-charge ratio for Tsp1a-IR800_P_ was successful and showed ions corresponding to the calculated mass of Tsp1a-IR800_P_. The calculated mass of Tsp1a-IR800_P_ was 4,682.87 for (3H)C_207_H_293_N_48_O_57_S_10_ and 1,563.03 for (3H)C_207_H_293_N_48_O_57_S_10_ [M+3H]^3+^, the found mass was 1,564.11 for C_207_H_293_N_48_O_57_S_10_ [M+3H]^3+^, the calculated mass was 1,172.20 for (3H)C_207_H_293_N_48_O_57_S_10_ [M+4H]^4+^, and the found mass was 1,173.02 for C_207_H_293_N_48_O_57_S_10_ [M+4H]^4+^. The HPLC retention time for the Tsp1a-IR800_P_ was 21.7 min (R_t_ = 21.7 min).

### Animal Work

All animal care and procedures were approved by the Animal Care and Use Committees of Memorial Sloan Kettering Cancer Center.

### Mouse Experiments

Hsd:athymic female mice (Nude-Foxn1^nu^, 6–8 wk old) were acquired from Jackson Laboratory. We first assessed the possibility of visualizing the olfactory nerve in normosmic mice using Tsp1a-IR800_P_. Nine mice were divided into 3 groups: experimental, control, and blocking. The experimental group was intravenously injected with Tsp1a-IR800_P_ (1 nmol in 100 μL of phosphate-buffered saline [PBS]), the control group was injected with 100 μL of PBS, and the blocking group was injected with a combination of Tsp1a-IR800_P_ and unmodified Tsp1a-Pra0 (1 nmol of Tsp1a-IR800_P_ plus 5 nmol of Tsp1a-Pra0 to form the blocking agent in 100 μL of PBS). Epifluorescence images were acquired 3 min after injection.

### Fluorescence Imaging of Mice

Fluorescence imaging was performed to identify fluorescence emitted by olfactory epithelium after injection of the compound. All mice were anesthetized using an intraperitoneal injection of a cocktail of 90 mg of ketamine and 10 mg of xylazine per kilogram, and in vivo epifluorescence images were obtained using an IVIS Spectrum imaging system (PerkinElmer) with filters set for 745-nm excitation and 800-, 820-, 840-nm emission. The animals were euthanized using CO_2_ asphyxiation, and olfactory nerve/bulb, muscle, heart, spleen, kidney, and liver were removed and imaged ex vivo using the IVIS Spectrum system. Autofluorescence was removed through spectral unmixing. Semiquantitative analysis of the Tsp1a-IR800_P_ signal was conducted by measuring the average radiant efficiency (in units of [p/s/cm^2^/sr]/[μW/cm^2^]) in regions of interest that were placed on the region of the olfactory bulb and nerve. Radiant efficiency was calculated using the following formula: radiant efficiency (p/s/cm^2^/sr)/(μW/cm^2^) = flux (photons/s)/excitation power (μW). Flux is the total photon flux emitted by the sample, which can be determined from the emission images. Excitation power is the power of the excitation light used for imaging.

Regarding mice infected with severe acute respiratory syndrome coronavirus 2 (SARS-CoV-2), the protocol has been described by Ordonez et al. (17). The supplemental materials provide details.

### Olfactory Ablation (Animal Model for Smell Disorders)

Methimazole treatment was performed to ablate the olfactory nerve as described by Bergman et al. (18). Mice were injected with methimazole dissolved in 0.9% saline (50 mg/kg of body weight) via intraperitoneal administration on days 0 and 3 from the start of treatment. In vivo, fluorescence imaging was performed on day 8. For this part of the experiment, we used 3 groups of mice, a group treated with methimazole (*n* = 3, 1 nmol in 100 μL of PBS), a normosmic group (*n* = 3, 1 nmol in 100 μL of PBS), and a blocking group (*n* = 3, normosmic mice injected with blocking agent in 100 μL of PBS). After imaging, all mice were euthanized, and organs of interest were dissected. Epifluorescence images were acquired 3 min after injection.

### Behavioral Experiment

The buried-food test was performed to prove the sense of smell impairment in olfactory-ablated mice. The buried-food test was performed as previously described (8). The supplemental materials provide more details.

### Dissection of Olfactory Bulb and Epithelium in Mice

Mouse olfactory bulb and epithelium were dissected to confirm our findings of the in vivo fluorescent experiments. Mice were anesthetized with ketamine and xylazine (100 and 20 μg/g of body weight, respectively) and perfused transcardially with 0.1 M phosphate buffer followed by a phosphate buffer fixative containing 4% paraformaldehyde as described by Lin et al. (19). The dissection was divided into 2 components: removal of the brain from the body and removal of the skin and lower jaw. Under ×2 loupe magnification, we removed the hard palate and the bones covering the brain, keeping nasal bones in place. The head was fixed in 4% paraformaldehyde overnight. Tissues were further processed, decalcified, and paraffin-embedded using a standard protocol (20).

### Nonmouse Animal Experiments

#### Hamsters

The protocol for hamsters has been described by Zazhytska et al. (3). The supplemental materials provide details.

#### Nonhuman Primates (NHPs)

Three NHPs (*Chlorocebus aethiops* [2 male and 1 female]) aged 5–8 y old were acquired from Worldwide Primates Inc. and allowed to acclimate in the vivarium for 3 mo. The animals were intravenously injected (brachial vein) with Tsp1a-IR800_P_ (200 μg/kg in 5 mL of 0.9% saline) and euthanized after 120 min using an intravenous barbiturate overdose (pentobarbital ≥ 150 mg/kg). Tissues of the olfactory epithelium, olfactory bulb, muscle, and frontal lobes of the brain were isolated and resected during necropsy. The tissues were further fixed in 4% paraformaldehyde for 12 h and imaged using the Quest imaging system.

### Fluorescence Imaging of NHP Tissues Using Clinically Available Fluorescent Camera

After intravenous injection of Tsp1a-IR800_P_, the animals were euthanized (using an intravenous barbiturate overdose) and tissues including olfactory epithelium, olfactory bulb, muscles, and brain were harvested. To image those tissues, we used a fluorescent camera that is clinically available (Quest Medical Imaging). The tissues were placed on top of nonreflective black paper to minimize reflection. The camera of the Quest near-infrared (NIR) imaging system was fixated 15 cm above the tissues. We used the same settings that are normally used for a typical imaging procedure (30-ms exposure time, 100% excitation power, and 25.5-dB gain, at 24 frames per second). Analysis and quantifications were performed on the QIFS research tool (Quest Medical Imaging). Regions of interest were drawn on the dissected tissues to determine differences.

### Methods Used to Work on Dissected Tissues

#### Immunohistochemistry and Quantification of Na_V_1.7 Expression

Na_V_1.7 staining was performed by the Molecular Cytology Core Facility of the Memorial Sloan Kettering Cancer Center using a Discovery XT processor (Ventana Medical Systems), according to our previously described protocol using anti-Na_V_1.7 antibody (N68/6; NeuroMab) that binds to both human and mouse Na_V_1.7 (0.5 μg/mL) (12). Na_V_1.7 quantification was performed on digitalized slides. The supplemental materials provide details.

#### Hematoxylin and Eosin Staining

We used a standard staining procedure. In short, tissue sections were deparaffinized in xylene and rehydrated through ethanol solutions. Hematoxylin was applied to stain nuclei, and after differentiation, eosin was used to stain cytoplasm. The slides were dehydrated with ascending ethanol concentrations, cleared with xylene, and mounted with a medium. After drying, the slides were examined under a microscope to visualize tissue structure and morphology.

### Obtaining Human Cadaveric Specimens

This study, conducted at Columbia University Irving Medical Center, involved 25 patients who had been previously diagnosed with COVID-19 by SARS-CoV-2 reverse transcription polymerase chain reaction analysis and underwent full-body autopsy. The study was approved by the Ethics and Institutional Review Board of Columbia University Medical Center (approvals AAAT0689 and AAAS7370). The supplemental materials provide details.

### Statistical Analysis

Statistical analysis of Na_V_1.7 expression was performed using R version 3.6.3 (R Core Team) and Prism 9 (GraphPad Software). The Student *t* test was used to compare Na_V_1.7 expression (ratio of Nav1.7 expression to total tissue area) and the fluorescent intensity difference between different groups of mice. A normal distribution of the variables was confirmed using the Shapiro–Wilk test. The Pearson correlation coefficient was used to examine the correlation between the time on the buried-food test and radiant efficiency. Results with a *P* value equal to or lower than 0.05 were considered statistically significant. Data points represent mean values, and error bars represent SD.

## RESULTS

### Na_V_1.7 Is Abundantly Expressed in Region of Olfactory Epithelium and Bulb (ROEB) of Normosmic Mice

**FIGURE 1.**
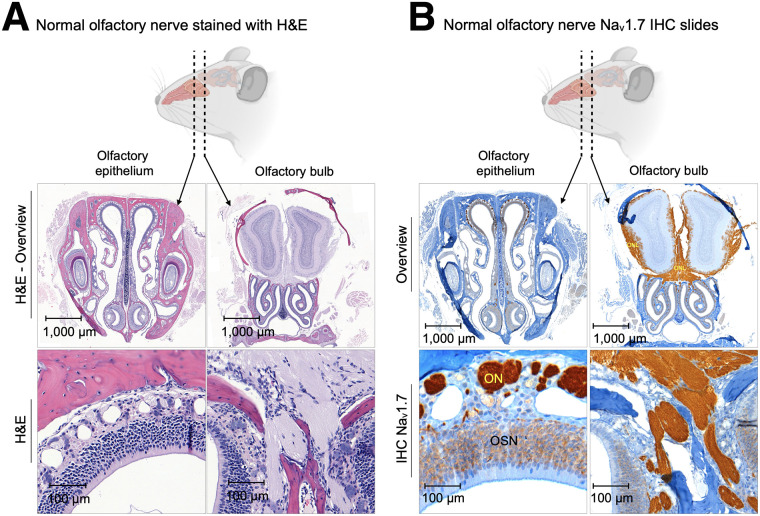
Histologic slides of olfactory bulb and olfactory epithelium of normosmic mice. (A) Hematoxylin and eosin staining. (B) Na_V_1.7 immunohistochemistry. H&E = hematoxylin and eosin; IHC = immunohistochemistry; ON = olfactory nerve bundles; ONL = olfactory nerve layer.

We isolated and dissected olfactory bulb and epithelium regions from normosmic nude mice and used immunohistochemistry (hematoxylin and eosin and anti-Na_V_1.7 antibody staining) to identify areas of high Na_V_1.7 expression. Consistent with previous reports (9*,*^10^), we found that Na_V_1.7 is moderately expressed in layers of primary OSNs and highly expressed in olfactory nerve bundles located in the lamina propria, including all the paths toward the olfactory bulb (Figs. 1A and 1B). The olfactory bulbs abundantly express Na_V_1.7 in peripheral areas, which correspond to the olfactory nerve layer. The glomerular layer, a layer deeper to the olfactory nerve layer, corresponds to terminal synapses of OSN axons, with the dendrites of mitral and tufted cells lightly stained for Na_V_1.7 (Fig. 1B).

### Na_V_1.7 Expression Is Downregulated in Chemically Induced Anosmia and COVID-19–Infected Mice

**FIGURE 2.**
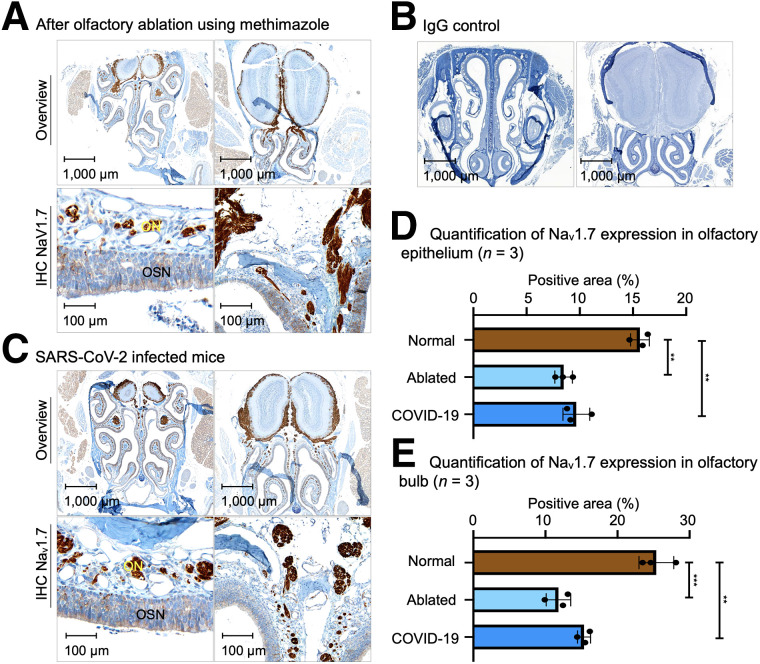
Histologic slides of olfactory bulb and olfactory epithelium of mice with olfactory ablation using methimazole and mouse infected with COVID-19. (A) Immunohistochemistry slide of mouse after olfactory ablation. (B) Immunohistochemistry slide of olfactory tissue with IgG isotype primary antibody, as control for possible unspecific binding. (C) Immunohistochemistry slides of mouse with SARS-CoV-2 infection. (D) Quantification of Na_V_1.7 expression in olfactory epithelium of 3 mouse groups. (E) Quantification of Na_V_1.7 expression in olfactory bulb of 3 mouse groups. ** *P* ≤ 0.01. *** *P* ≤ 0.001. IHC = immunohistochemistry; ON = olfactory nerve bundles; ONL = olfactory nerve layer.

Our immunohistochemistry data suggest that the expression of Na_V_1.7 was significantly diminished after olfactory ablation using methimazole and in SARS-CoV-2–infected mice (Figs. 2A–2E). Of the total tissue area in the olfactory epithelium of normosmic mice, 15.7% (±0.83%) was positive for Na_V_1.7 expression, compared with 8.5% (±0.94%) in mice that had their olfactory sense ablated by methimazole and 9.7% (±1.2%) in mice infected with SARS-CoV-2 (Fig. 2D, *P* ≤ 0.01). The olfactory nerve layer of the olfactory bulb also showed a decrease in Na_V_1.7 expression in both olfactory-ablated and SARS-CoV-2 infected mice (Fig. 2E). Normosmic mice have a 25.4% (±2.4%) positive Na_V_1.7 area compared with 11.8% (±1.7%) in ablated and 15.4% (±0.9%) in SARS-CoV-2–infected mice (Fig. 2E).

### RNA Sequencing Reveals Temporal Downregulation of SCN9A Gene Expression in OSN Cells from Hamsters Infected with SARS-CoV-2 and Correlates with Loss and Gain of Olfactory Function

**FIGURE 3.**
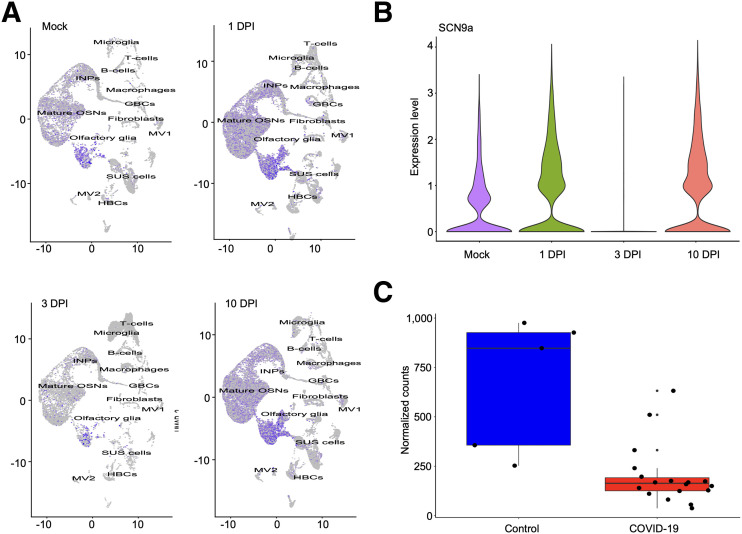
SCN9A gene expression in olfactory epithelium of hamsters and humans infected with SARS-CoV-2. (A) Uniform manifold approximation and projection for dimension reduction plots of SCN9A gene expression in different cell types of olfactory epithelium in mock and SARS-CoV-2–infected hamsters at 1, 3, and 10 d after infection. (B) Violin plots of SCN9A gene expression in olfactory epithelium bulk tissues in mock and SARS-CoV-2–infected hamsters at 1, 3, 10 d after infection. (C) SCN9A gene expression in human olfactory epithelium tissues in control and SARS-CoV-2–infected cadavers. DPI = days after infection; GBC = glucose basal cells; HBC = horizontal basal cells; INP = immediate neuronal precursors; MV2 = microvillus cells 2; OSN = olfactory sensory neurons; SUS = sustentacular cells.

We performed bulk and single-cell RNA sequencing of SARS-CoV-2–infected and mock hamsters’ olfactory epithelium tissues at 1, 3, and 10 d after infection. For single-cell RNA sequencing, we analyzed 68,951 cells and identified 13 cell subtypes using previously described markers (21). SCN9A was expressed predominantly in OSNs and olfactory glia, with minimal to zero expression in other cell subtypes in the olfactory epithelium. In SARS-CoV-2–infected hamsters, we observed a 3-fold drop in expression of the SCN9A gene transcripts in OSNs at 3 d after infection and full recovery of expression at 10 d after infection with bulk RNA sequencing analysis (*P* < 0.001). These changes in SCN9A transcripts can be followed on Uniform Manifold Approximation and Projection for Dimension Reduction clustering maps that show temporal downregulation of SCN9A. At 3 d after infection, the transcript levels were significantly diminished compared with the control and first day after infection, and at 10 d after infection, they were restored to preinfection levels, as is clearly visible in the figures (Figs. 3A and 3B; Supplemental Fig. 2A).

### SARS-CoV-2 Infection Induces Downregulation of SCN9A Gene in Human OSN

To verify whether changes in SCN9A expression in ROEB observed in COVID-19–infected mice and hamsters were also observed in humans, we tested the Na_V_1.7 expression in the OSNs of cadavers. The region of the cribriform plate, located at the roof of the nasal cavity, in 23 human cadavers—18 COVID-19–infected and 5 controls—was dissected. This region has a high density of mature OSNs and therefore is suitable for the experimental plan. We then performed bulk RNA sequencing of olfactory epithelium tissues from the resected specimens. Cadavers were donated from patients of different sexes (9 male and 14 female), with the median age of patients being 73 y (interquartile range, 65–78 y), representing a variety of infection durations, hospital stays, treatments, and postmortem intervals. We have previously demonstrated that despite different postmortem times for collecting the samples, only a minimal influence on the cellular constitution of tissues and immune cells in the olfactory epithelium of humans could be observed (3). Therefore, we extrapolated this for the Na_V_1.7 expression. SARS-CoV-2 was detected in all positive olfactory epithelium tissues with variations in the viral load (3). SCN9A gene expression is 4-fold lower in SARS-CoV-2–infected olfactory epithelium tissue samples than in healthy controls (*P* < 0.001) (Fig. 3C; Supplemental Fig. 2B).

### Na_V_1.7 Expression in ROEB of Healthy Mice Can Be Imaged Using Tsp1a-IR800_P_

**FIGURE 4.**
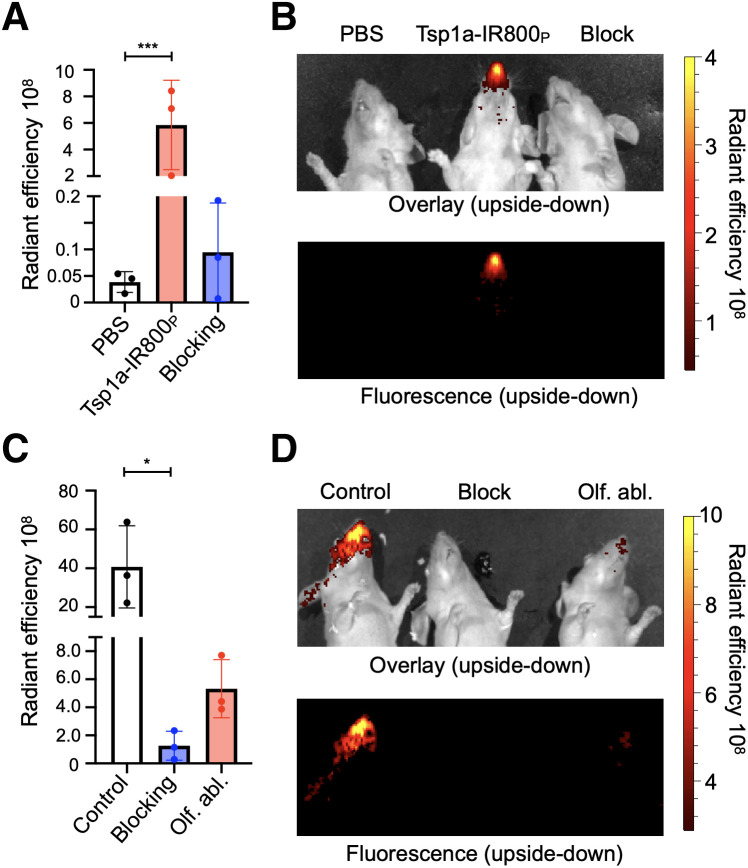
Tsp1a-IR800_P_ accumulation in ROEB in normosmic mice and mice with olfactory ablation. (A and B) Fluorescence quantification and epifluorescence images of animals injected with PBS, Tsp1a-IR800_P_, and Tsp1a-IR800_P_/Tsp1a blocking formulation. (C and D) Epifluorescence images and fluorescence intensity quantification of normosmic animals injected with Tsp1a-IR800_P_ (control) and Tsp1a-IR800_P_/Tsp1a (blocking) and mice with prior olfactory ablation using methimazole injected with Tsp1a-IR800_P_. All images were taken 30 min after tail vein injection. *P ≤ 0.05. ***P ≤ 0.001. Olf. abl. = olfactory ablation.

Using a widely available IVIS Spectrum imaging system, we could clearly visualize Na_V_1.7 expression in mouse ROEB using fluorescence imaging without the need for any surgical intervention. Excitation was set to 745 nm, and emission was set to 800, 820, and 840 nm. We designed our probe to have these features because there is minimal background at these wavelengths, thus providing a highly specific signal. Because of the superficial expression of Na_V_1.7 in the mouse ROEB, we were able to obtain images of the mice without the need to expose the olfactory epithelium. Epifluorescence in vivo images in mice receiving intravenous injection with the imaging agent Tsp1a-IR800_P_ generated high contrast between the ROEB and its surrounding regions. The radiant efficiency was significantly less in both mice injected with PBS and mice injected with the unmodified peptide (blocking agent) in combination with the imaging agent (Figs. 4A and 4B). We observed a 150-fold ([5.9 ± 3.4] × 10^8^ vs. [3.9 ± 1.9] × 10^6^ [p/s/cm^2^/sr]/[μW/cm^2^], *P* < 0.0001) increase in radiant efficiency compared with mice injected with PBS. To demonstrate specificity, we coadministered our imaging probe with a non–fluorophore-labeled Tsp1a and observed a 61-fold decrease in signal emanating from the mouse olfactory epithelium.

### Tsp1a-IR800_P_ Can Differentiate Healthy from Anosmic Mice Through Its Fluorescence Emission

The group of mice that were treated with methimazole, widely known to cause damage to the olfactory nerves of those animals, had an 8-fold decrease in radiant efficiency signal compared with normosmic control mice when imaged with Tsp1a-IR800_P_ (Figs. 4C and 4D). The average radiant efficiency of the olfactory region of normosmic mice imaged from both sides was (4.08 ± 2.11) × 10^9^, compared with (5.33 ± 2.08) × 10^8^ for mice with an olfactory ablation (unpaired *t* test, *P* = 0.045). Ex vivo images showed a similar statistically significant difference between mouse groups in the ROEB. The heart and kidneys were the only internal organs with higher fluorescence signals than in normosmic mice and mice treated with a blocking agent.

### Tsp1a-IR800_P_ Shows Fluorescence Signals in OSN

**FIGURE 5.**
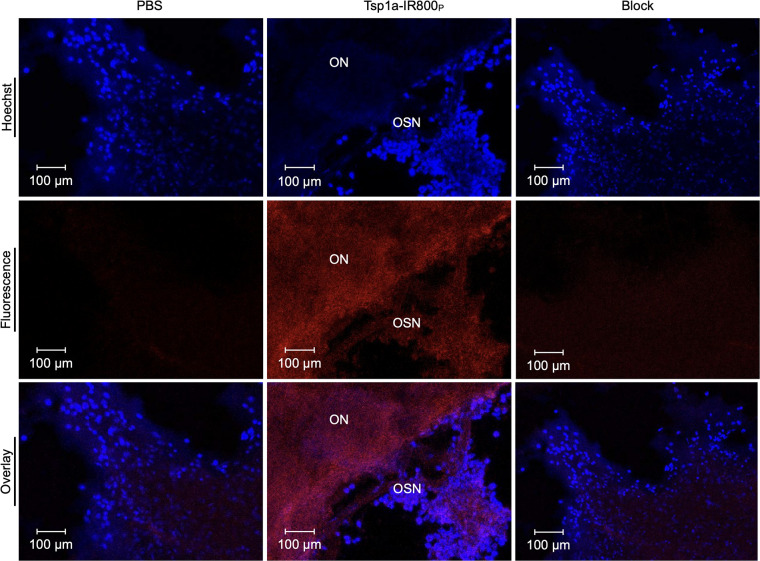
Fluorescent confocal microscopy images of olfactory epithelium of animals injected with PBS, Tsp1a-IR800_P_, and Tsp1a-IR800_P_/Tsp1a-Pra0 blocking formulation. Blue fluorescence indicates nucleus of cells, and red fluorescence indicates infrared fluorescence coming from Na_V_1.7 of olfactory nerve bundles. ON = olfactory nerve (bundles).

For further confirmation, we resected the olfactory epithelium region of these mice and obtained regular tabletop fluorescent microscopy images of sectioned tissue (Fig. 5). The fluorescent microscopy images confirmed the results of in vivo epifluorescence imaging and immunohistochemistry. OSN and olfactory nerve bundles located in the lamina propria showed the most intense fluorescence. Normosmic mice and mice treated with a blocking agent were negative for any signal (red staining), whereas only nucleus-associated blue staining was visible corresponding to nuclear staining by Hoechst dye.

### Time to Completion of Buried-Food Test Correlates with ROEB Fluorescence Emissions

**FIGURE 6.**
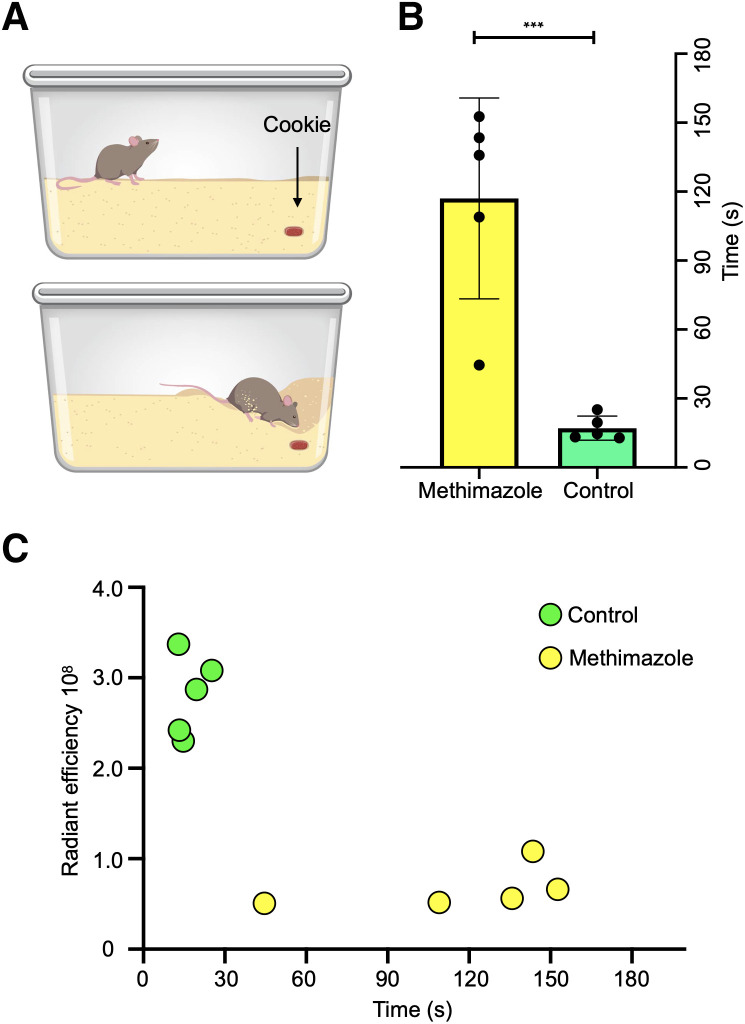
Buried-food test. (A) Schematic of experimental design illustrating mouse cage with cookie buried in upper right corner. (B) Average time (seconds) spent per mouse treated with PBS (*n* = 5) or methimazole (*n* = 5) to find buried food. Graph indicates that healthy mice found buried food much more quickly than ones treated with methimazole (*P* < 0.001), suggesting presence of olfactory dysfunction in the latter. (C) Correlation of Tsp1a-IR800_P_ radiant efficiency at ROEB and time on buried-food test demonstrating that the more quickly mice find buried food, the brighter is fluorescence detected from olfactory nerve region.

Normosmic mice were able to find buried food in less than 30 s (mean, 17 ± 5.2 s), compared with a mean time of 117 s (±44 s) for olfactory-ablated mice, showing that mice with olfactory ablation needed a significantly longer time (*P* < 0.001) than normosmic mice to find the buried food (Figs. 6A and 6B). Furthermore, there was an inverse correlation between the Tsp1a-IR800_P_ radiant efficiency and the time required to find buried food (*r* = –0.79, *n* = 10, *P* = 0.006; Fig. 6C).

### Na_V_1.7 Expression in Olfactory Epithelium of NHPs Can Be Detected Using Tsp1a-IR800_P_ and Clinically Approved NIR Camera

**FIGURE 7.**
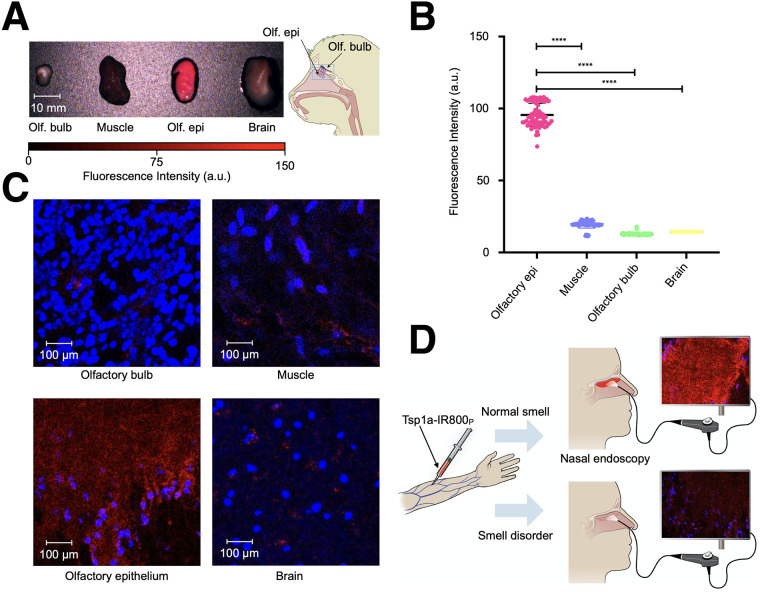
Imaging olfactory epithelium of NHPs. (A) Images were taken using Quest system (approved for clinical use) of olfactory bulb, muscle, olfactory epithelium, and brain of 4 NHPs, after intravenous injection of Tsp1a-IR800_P_. (B) Quantification of near-fluorescence intensity demonstrates that signal from olfactory epithelium is significantly higher than that from surrounding tissues. (C) Fluorescent confocal microscopy images of olfactory bulb, muscle, olfactory epithelium, and brain tissues of same NHPs. (D) Schematic depiction of potential use of Tsp1a-IR800_P_ in physician’s office setting using Quest or other vendor NIR fluorescence imaging systems. ****P ≤ 0.0001. a.u. = arbitrary units; olf. bulb = olfactory bulb; olf. epi = olfactory epithelium.

To validate the clinical potential of our approach of using Na_V_1.7 expression as a surrogate marker for the sense of smell, we imaged NHP specimens using a clinically applied Food and Drug Administration–approved commercial Quest NIR system. The bright fluorescence was clearly visible over the olfactory epithelium, and weak or no fluorescence was detected over the muscle, olfactory bulb, and brain. The fluorescence intensity of olfactory epithelium was significantly higher (*P* < 0.001) than that of any other measured tissue (Figs. 7A and 7B).

### Confocal Microscopy of NHP Tissues Corroborates Results from Clinically Approved NIR Device

To confirm our macroscopic results, we also performed fluorescence microscopy on the dissected NHP tissues (Fig. 7C). The fluorescence signal from the NHP olfactory epithelium was clearly brighter than that from other tissues. It is important to stress that the olfactory bulb expresses Na_V_1.7 but that the blood–brain barrier prevents entry of our imaging agent in the current setting. For the current application, this significant advantage reduces the potential background signal from the olfactory bulb, which could be immediately applicable to the clinical setting (Fig. 7D).

## DISCUSSION

We describe the development of a novel semiquantitative diagnostic method based on selective targeting of Na_V_1.7 expression in olfactory epithelium for the detection of smell disorders induced by different insults and inflammatory conditions such as COVID-19. The fluorescence signal emanating from mouse olfactory epithelium shows decreased intensity when mice have anosmia. We observed an inverse linear relationship between signal intensity and the degree of damage. Therefore, we believe that the described method has the potential to serve as an objective guide for assessing disease progression and treatment response both in animal and in human subjects and to aid drug development. We hypothesize that the absence of fluorescence may indicate a low probability of recovery of the smell and that fluorescence intensity restoration may be an early indicator of treatment response, preceding perceptible or clinical smell restoration. This is a significant development because the current tests are subjective by design and methods are culturally specific and individually subjective (22). In addition, current methods rely on invasive biopsy, which may deter patients and limit the number of times testing can be performed. Our method is noninvasive, can be performed repeatedly on animal subjects, and therefore has the potential to detect the posttreatment restoration process before improvement in the sense of smell can be noticed (23).

We have found that smell loss due to chronic inflammation in the nasal cavity or viral infection is accompanied by diminished expression of Na_V_1.7 in OSNs. We observed loss of Na_V_1.7 expression after olfactory ablation using methimazole (Fig. 2). Expression of Na_V_1.7 was substantially reduced in OSNs and in nerve bundles located in the lamina propria, whereas expression was less affected at the level of the olfactory bulbs. This probably happens because of the location of the olfactory bulbs—much deeper in the tissues and protected by the blood–brain barrier and cribriform plate.

Further, we assessed whether COVID-19–related olfaction loss is also accompanied by loss of Na_V_1.7. COVID-19–related smell disorders are possibly caused by multiple mechanisms. There is a direct tropism of the virus to sustentacular and microvillar cells covering OSNs; inflammatory damage to OSNs, as they are exposed to environmental factors; and inflammation involving focal mucosal swelling and obstruction to airways (24). Recently, our collaborators (Lomvardas Lab at Columbia University) reported that the downregulation in odor detection pathways could be a potential cause of COVID-19–induced anosmia (3). We found that these mechanisms cause diminished Na_V_1.7 expression in OSNs and olfactory nerve bundles located in the nasal cavity as shown by immunohistochemistry and RNA sequencing on tissues from mice, hamsters, and humans.

Our compound Tsp1a-IR800_P_ selectively binds to Na_V_1.7, making it an ideal candidate for measuring smell perception using fluorescent measurement techniques. We tested our imaging agent using a mouse model in which olfactory ablation via methimazole injections (25) led to reduced fluorescence in mouse nasal epithelium. This damage follows a sensorineural smell loss pattern similar to that seen in upper-respiratory viral infection or chronic inflammation due to seasonal allergies (16*,*^26^). Furthermore, we demonstrated that the radiant efficiency of the Na_V_1.7 imaging agent correlated inversely with the time that mice spent finding food in a buried-food test. These studies indicate that Tsp1a-IR800_P_ uptake inversely correlates with loss of Na_V_1.7 expression and therefore with loss of the sense of smell.

The difference between rodents and humans can be substantial, and to validate the possibility that olfaction can be imaged in humans, we tested our compound on healthy NHPs and measured fluorescence using a clinically approved NIR imaging system. We observed bright fluorescence from the olfactory epithelium, and the fluorescence measurements were significantly higher than in surrounding tissues.

It is crucial to develop a noninvasive, fast, and objective method to diagnose smell disorders. Intravenously injected Tsp1a-IR800_P_ selectively accumulates in OSNs and olfactory nerve bundles located in the nasal cavity. For the experiment, we used a clinically applied camera that can detect infrared wavelength fluorescence using a rigid endoscope. Existing flexible endoscopes from several medical technology companies (Quest, Stryker, Olympus, etc.) can detect the infrared wavelength emitted by Tsp1a-IR800_P_ (Fig. 7D). Because of the minimally invasive nature of the nasal cavity endoscope, and the correlation of fluorescence with the magnitude of smell loss, the method can be used as a treatment-monitoring tool and in drug development. In addition, the method can be widely used in an experimental setting to objectively measure smell in animals, replacing the need to perform behavioral experiments and streamlining the development of new therapeutics. An obvious limitation of the technique is that if loss of olfaction is a result of olfactory receptor or olfactory bulb dysfunction, the applicability of our imaging technique might be limited, though further studies are needed on the influence of such dysfunction on Na_V_1.7 expression in OSNs.

## CONCLUSION

The described method of applying the novel fluorescent probe Tsp1a-IR800_P_ could help significantly improve preclinical and clinical studies by providing an objective way to diagnose smell disorders and aid in the development of therapeutic interventions.

## DISCLOSURE

This work was supported by National Institutes of Health grants R01 EB029769 (Nagavarakishore Pillarsetty and Glenn King), R01 CA204441 (Thomas Reiner), K99 GM145587 (Junior Gonzales), and R01 CA204441-03S1 (Junior Gonzales); the Australian National Health and Medical Research Council (principal research fellowship APP1136889 to Glenn King); and the Australia Research Council (Centre of Excellence grant CE200100012 to Glenn King). Support is acknowledged from Mr. William H. Goodwin and Mrs. Alice Goodwin and the Commonwealth Foundation for Cancer Research and the Center for Experimental Therapeutics of Memorial Sloan Kettering Cancer Center. The funding sources were not involved in study design, data collection and analysis, writing of the report, or the decision to submit this article for publication. Funding from Memorial Sloan Kettering Cancer Center grant P30 CA008748 for core facility support is also acknowledged. Snehal Patel and Thomas Reiner are shareholders of Summit Biomedical Imaging. Thomas Reiner is now an employee of Evergreen Theragnostics. Junior Gonzales, Paula Demetrio de Souza Franca, Glenn King, and Thomas Reiner are coinventors on a Tsp1a-related patent application. No other potential conflict of interest relevant to this article was reported.

## Notes

KEY POINTS **QUESTION:** Is it possible to image the olfactory nerve and diagnose smell disorders by a molecularly targeted (Na_V_1.7) fluorescent agent? **PERTINENT FINDINGS:** In this animal study, we have shown that several causal factors of smell disorders, such as chronic inflammation, allergy, and viral infection including COVID-19, result in substantially diminished expression of Na_V_1.7 at the transcript and protein levels. We have shown that a new fluorescently labeled agent, Tsp1a-IR800_P_, molecularly targets Na_V_1.7 and can be used to semiquantitatively measure olfaction and diagnose smell disorders in animals and humans. **IMPLICATIONS FOR PATIENT CARE:** To study smell and smell disorders, there will be no need for behavioral experiments on animals. Furthermore, smell disorders such as anosmia in humans potentially can be diagnosed in a physician’s office setting using our agent and endoscope.

## References

[1] Asahina K Pavlenkovich V Vosshall LB. The survival advantage of olfaction in a competitive environment. Curr Biol. 2008;18:1153–1155. 18674910 10.1016/j.cub.2008.06.075 PMC2575080

[2] Nalbandian A Sehgal K Gupta A. Post-acute COVID-19 syndrome. Nat Med. 2021;27:601–615. 33753937 10.1038/s41591-021-01283-z PMC8893149

[3] Zazhytska M Kodra A Hoagland DA. Non-cell-autonomous disruption of nuclear architecture as a potential cause of COVID-19-induced anosmia. Cell. 2022;185:1052–1064.e12. 10.1016/j.cell.2022.01.024 PMC8808699 35180380

[4] Hoffman HJ Rawal S Li CM Duffy VB. New chemosensory component in the US National Health and Nutrition Examination Survey (NHANES): first-year results for measured olfactory dysfunction. Rev Endocr Metab Disord. 2016;17:221–240. 27287364 10.1007/s11154-016-9364-1 PMC5033684

[5] Burges Watson DL Campbell M Hopkins C Smith B Kelly C Deary V. Altered smell and taste: anosmia, parosmia and the impact of long Covid-19. PLoS One. 2021;16:e0256998. 34559820 10.1371/journal.pone.0256998 PMC8462678

[6] Mendes Paranhos AC Nazareth Dias AR Machado da Silva LC. Sociodemographic characteristics and comorbidities of patients with long COVID and persistent olfactory dysfunction. JAMA Netw Open. 2022;5:e2230637. 36074464 10.1001/jamanetworkopen.2022.30637 PMC9459661

[7] Boscolo-Rizzo P Tirelli G Meloni P. Coronavirus disease 2019 (COVID-19)-related smell and taste impairment with widespread diffusion of severe acute respiratory syndrome-coronavirus-2 (SARS-CoV-2) omicron variant. Int Forum Allergy Rhinol. 2022;12:1273–1281. 35286777 10.1002/alr.22995 PMC9082058

[8] Yang M Crawley JN. Simple behavioral assessment of mouse olfaction. Curr Protoc Neurosci. 2009;48:8.24.1–8.24.12. 10.1002/0471142301.ns0824s48 PMC2753229 19575474

[9] Rupasinghe DB Knapp O Blomster LV. Localization of Nav1.7 in the normal and injured rodent olfactory system indicates a critical role in olfaction, pheromone sensing and immune function. Channels. 2012;6:103–110. 22622154 10.4161/chan.19484

[10] Weiss J Pyrski M Jacobi E. Loss-of-function mutations in sodium channel Na_v_1.7 cause anosmia. Nature. 2011;472:186–190. 21441906 10.1038/nature09975 PMC3674497

[11] Jiang Y Castro J Blomster LV. Pharmacological inhibition of the voltage-gated sodium channel Na_V_1.7 alleviates chronic visceral pain in a rodent model of irritable bowel syndrome. ACS Pharmacol Transl Sci. 2021;4:1362–1378. 34423271 10.1021/acsptsci.1c00072 PMC8369682

[12] Gonzales J Franca PDD Jiang Y. Fluorescence imaging of peripheral nerves by a Na_v_1.7-targeted inhibitor cystine knot peptide. Bioconjug Chem. 2019;30:2879–2888. 31647222 10.1021/acs.bioconjchem.9b00612 PMC7372312

[13] Gonzales J Adilbay D. Franca PDdS, et al. NaV1.7 targeted fluorescence imaging agents for nerve identification during intraoperative procedures. bioRxiv website. https://www.biorxiv.org/content/10.1101/2024.04.06.588368v1. Published April 6, 2024. Accessed June 4, 2024.

[14] Gonzales J Hernández-Gil J Wilson TC. Bimodal imaging of mouse peripheral nerves with chlorin tracers. Mol Pharm. 2021;18:940–951. 33404254 10.1021/acs.molpharmaceut.0c00946 PMC7920913

[15] Hernández-Gil J Chow CY Chatras H. Development and validation of nerve-targeted bacteriochlorin sensors. J Am Chem Soc. 2023;145:14276–14287. 37339504 10.1021/jacs.3c02520 PMC11443384

[16] Håglin S Bohm S Berghard A. Single or repeated ablation of mouse olfactory epithelium by methimazole. Bio Protoc. 2021;11:e3983. 10.21769/BioProtoc.3983 PMC8160539 34124287

[17] Ordonez AA Bullen CK Villabona-Rueda AF. Sulforaphane exhibits antiviral activity against pandemic SARS-CoV-2 and seasonal HCoV-OC43 coronaviruses in vitro and in mice. Commun Biol. 2022;5:242. 10.1038/s42003-022-03189-z PMC8933402 35304580

[18] Bergman U Ostergren A Gustafson AL Brittebo EB. Differential effects of olfactory toxicants on olfactory regeneration. Arch Toxicol. 2002;76:104–112. 11914780 10.1007/s00204-002-0321-2

[19] Lin W Ogura T Margolskee RF Finger TE Restrepo D. TRPM5-expressing solitary chemosensory cells respond to odorous irritants. J Neurophysiol. 2008;99:1451–1460. 18160424 10.1152/jn.01195.2007

[20] Alaeddini M Bashizadehfakhar H Amirinia F. The effect of different combinations of fixatives and decalcifying agents on rat and rabbit hard tissues, a guide for histologic processing. Acta Histochem. 2022;124:151962. 10.1016/j.acthis.2022.151962 36228481

[21] Durante MA Kurtenbach S Sargi ZB. Single-cell analysis of olfactory neurogenesis and differentiation in adult humans. Nat Neurosci. 2020;23:323–326. 32066986 10.1038/s41593-020-0587-9 PMC7065961

[22] Hummel T Whitcroft KL Andrews P. Position paper on olfactory dysfunction. Rhinology. 2016;56:1–30. 28623665 10.4193/Rhino16.248

[23] Mainland JD Barlow LA Munger SD. Identifying treatments for taste and smell disorders: gaps and opportunities. Chem Senses. 2020;45:493–502. 32556127 10.1093/chemse/bjaa038 PMC7545248

[24] Mastrangelo A Bonato M Cinque P. Smell and taste disorders in COVID-19: from pathogenesis to clinical features and outcomes. Neurosci Lett. 2021;748:135694. 33600902 10.1016/j.neulet.2021.135694 PMC7883672

[25] Bergström U Giovanetti A Piras E Brittebo EB. Methimazole-induced damage in the olfactory mucosa: effects on ultrastructure and glutathione levels. Toxicol Pathol. 2003;31:379–387. 12851103 10.1080/01926230390201101

[26] Jung AY Kim YH. Reversal of olfactory disturbance in allergic rhinitis related to OMP suppression by intranasal budesonide treatment. Allergy Asthma Immunol Res. 2020;12:110–124. 31743968 10.4168/aair.2020.12.1.110 PMC6875474

